# Transcriptomic response analysis of ultraviolet mutagenesis combined with high carbon acclimation to promote photosynthetic carbon assimilation in *Euglena gracilis*

**DOI:** 10.3389/fmicb.2024.1444420

**Published:** 2024-08-29

**Authors:** Qi Lv, Siping Li, Xinxin Du, Yawen Fan, Mingshuo Wang, Chunhua Song, Fengyang Sui, Yan Liu

**Affiliations:** ^1^College of Life Sciences and Technology, Harbin Normal University, Harbin, China; ^2^Key Laboratory of Biodiversity of Aquatic Organisms, Harbin Normal University, Harbin, China

**Keywords:** *Euglena gracilis*, mutant, efficient carbon sequestration, physiological response, transcriptome

## Abstract

The potential of *Euglena gracilis* for carbon sequestration offers significant opportunities in the capture and utilization of carbon dioxide (CO_2_). In this study, a mutant LE-ZW of *E. gracilis*, capable of efficient growth and carbon sequestration, was obtained through ultraviolet mutagenesis combined with high carbon acclimation. Subsequently, the potential of LE-ZW for carbon assimilation was systematically analyzed. The results demonstrated that the cell density of the LE-ZW was 1.33 times that of the wild type and its carbon sequestration efficiency was 6.67 times that of the wild type when cultured at an optimal CO_2_ concentration of 5% until day 10. At this time, most key enzyme genes associated with the photosystem membrane protein complex, photosynthetic electron transport chain, antenna protein, and carbon fixation were up-regulated in mutant LE-ZW. Furthermore, after 10 days of culture under 10% CO_2_, the cell density and carbon sequestration efficiency of LE-ZW reached 1.10 times and 1.54 times of that under 5% CO_2_, respectively. Transcriptome analysis revealed significant up-regulation of key enzyme genes associated with carbon fixation, central carbon metabolism, and photosynthesis in LE-ZW under a 10% CO_2_ concentration. Physiological indices such as the amount of oxygen evolution, the values of Fv/Fm, the expression levels of photosynthetic protein genes and the enzyme activity of key enzymes related to photosynthetic carbon assimilation were corroborated by transcriptome data, elucidating that the mutant LE-ZW exhibited augmented photosynthetic carbon sequestration capacity and metabolic activity, thereby demonstrating robust adaptability to a high-carbon environment. This research contributes to a deeper understanding of the carbon assimilation mechanism in photosynthetic protists under elevated CO_2_ concentrations.

## 1 Introduction

The issue of global warming has gained significant attention, leading to commitments from over 130 countries and regions worldwide to achieve carbon neutrality. Consequently, the momentum towards the development of a “low-carbon economy” has been steadily increasing (Jiao et al., 2024). Various physical, chemical, and biological methods have been proposed to tackle the challenge of carbon capture, utilization, and storage (CCUS) (Hou et al., 2024). Microalgae, as microscopic photosynthetic autotrophs, exhibit remarkable carbon sequestration capabilities and are regarded as a novel “promising tool” for mitigating carbon emissions (Khan et al., 2018). Therefore, utilizing microalgae for carbon sequestration has become a cutting-edge research focus and a key area of competition in international emission reduction and new energy development. The increase of CO_2_ concentration in the environment within the range of physiological adaptation of microalgae species can effectively improve the growth rate and photosynthetic efficiency of microalgae to a certain extent, and then promote the improvement of microalgae biomass (Gharanjik et al., 2024). However, the higher the carbon concentration, the higher the carbon sequestration efficiency of microalgae is not necessarily, and the optimal CO_2_ concentration is 0.038–10% for most microalgae (Yaqi et al., 2023). Nevertheless, the concentration of CO_2_ in industrial flue gas can reach as high as 10–20%, which may impede the growth of microalgae and diminish the efficiency of carbon sequestration (Li et al., 2015). Microalgal growth is typically significantly inhibited at CO_2_ concentrations exceeding 10%, probably due to the low pH caused by high CO_2_ levels (Inwongwan et al., 2019). Therefore, the utilization of microalgae for mitigating flue gas CO_2_ emissions necessitates the meticulous selection of algae strains capable of enduring elevated CO_2_ concentrations. Such algal strains should possess a robust CO_2_ tolerance, exhibit a high level of enzyme activity to achieve an elevated carbon sequestration rate, be amenable to large-scale cultivation, and contain valuable components with high added value.

*Euglena gracilis,* a unicellular eukaryotic microalga devoid of cell walls, belongs to the Euglenophyta within the kingdom Protozoa. *E. gracilis* exhibits both animal-like and plant-like characteristics, possessing chlorophyll for photosynthesis while also capable of heterotrophic survival in light-deprived conditions (Edmunds, 1965). Due to this characteristic, several studies have proposed its classification as a unicellular flagellate, belongs to the Euglenida (Ebenezer et al., 2022). The *E. gracilis* is widely recognized as a paradigmatic organism and has been extensively employed in fundamental ecological and physiological investigations. In addition, as a model organism, *E. gracilis* has achieved large-scale industrial production (Susumu et al., 2020). Studies have demonstrated that *E. gracilis* is abundant in assimilative products, such as paramylon, protein, various unsaturated fatty acids, tocopherols, and 59 other essential nutrients for human health (Mou et al., 2024; Fukuda et al., 2024; Huang et al., 2023; Mokrosnop et al., 2016). In the face of climate change, *E. gracilis* has exhibited an outstanding capacity for capturing CO_2_. It has been reported that the optimal growth of *E. gracilis* occurs under a CO_2_ concentration of 5%. Subsequently, as the CO_2_ concentration gradually increases, both their growth rate and carbon sequestration ability diminish. Nevertheless, compared to other protist species, *E. gracilis* exhibits remarkable tolerance to elevated levels of CO_2_, growing and reproducing even at concentrations as high as 40% (Nakano et al., 1996).

The previous experimental study conducted by our research group has also confirmed that *E. gracilis* exhibits optimal growth when ventilated to a CO_2_ concentration of 5% (Anlong et al., 2023). However, to efficiently utilize *E. gracilis* for fixing industrial flue gas and realizing CO_2_ resource utilization, higher adaptability under high CO_2_ conditions needs to be emphasized. Therefore, in this study, a mutant strain of *E. gracilis* named LE-ZW was obtained through ultraviolet (UV) mutagenesis and high carbon acclimation, exhibiting enhanced growth and carbon sequestration capabilities under a high CO_2_ concentration of 10%. In order to explore the promotion effect of UV mutagenesis combined with high-carbon acclimation on the photosynthetic carbon assimilation ability of *E. gracilis*, transcriptomic data combined with physiological indicators were used to investigate the key factors of the mutant’s response to elevated CO_2_ at a genetic level. This study provides targeted molecular and physiological explanations for the evolution of carbon sequestration ability of the mutant of *E. gracilis*, expands the understanding of key metabolic and biological pathways in *E. gracilis* under high CO_2_ levels, and enhances the engineering application potential of carbon sequestration by *E. gracilis*.

## 2 Materials and methods

### 2.1 Experimental algae species acquisition and culture

The strain of *E. gracilis* was provided by the research group of Professor Wang Jiangxin at the College of Life Sciences and Oceanography, Shenzhen University. *E. gracilis* was cultured in a sterile light incubator using AF-6 medium at a culture temperature of 25 ± 1°C, illuminance of 6,000 lx, and light-to-darkness ratio of 15 L: 9D.

### 2.2 UV mutagenesis and high carbon acclimation

#### 2.2.1 UV mutagenesis

The wild-type strain was mutated by the UV lamp. The logarithmic growth stage of *E. gracilis* was selected, diluted to a concentration of 10^5^ cells/mL, and evenly coated on AGAR plates containing AF-6 medium. Subsequently, position the samples at distances of 15 cm and 25 cm from the UV lamp, ensuring a UV light intensity of 20 watts and an irradiation wavelength of 254 nm. A radiation exposure time gradient of 0 min (as a control), 5 min, 10 min, 15 min, 20 min, and 25 min was established. After irradiation, the samples were kept in the dark for 24 h to prevent light repair. After 1 d, the light recovery cultures were performed in the light incubator, and the mortality rate of *E. gracilis* after mutagenesis was analyzed (Huixia et al., 2019). The lethality rate (LR) equation for this study was shown in.

LR (%) = (C_0_ - C_i_)/C_0_ × 100%.

where C_0_ represents the average number of single algal colonies in control group on AGAR medium, and C_i_ represents the average number of single algal colonies in experimental group on AGAR medium.

According to previous study, when the fatality rate is between 80 and 90%, the probability of forward mutation being realized is higher (Yanxia, 2021). Dark green and large algal colonies with a mutagenesis fatality rate of 80% ~ 90% were selected and inoculated in liquid medium for subsequent monoclonal culture. Cell density was prioritized as the screening indicator, several mutants of *E. gracilis* with excellent growth were initially screened out.

#### 2.2.2 High carbon acclimation

Each CO_2_ concentration (5, 6, 7, 8, 9, and 10%) was set as an acclimation cycle, the acclimation time was 6 cycles, and one cycle was 7 days, and the gas (V_CO2_/ V_air_) ventilation rate was 300 mL·min^−1^. After acclimation, the growth of each strain was tested under 10% CO_2_, with the wild-type *E. gracilis* strain serving as a control. The cell density, chlorophyll a content, and chlorophyll b content were selected as the priority screening indicators to select the strain with the best growth and the highest photosynthetic pigment content, which was named LE-ZW for further experiments.

### 2.3 Experimental design for investigating the photosynthetic carbon assimilation characteristics of LE-ZW

Three experimental groups were set up: CK (the wild-type of *E. gracilis* strain was injected with 5% CO_2_), ZW1 (the LE-ZW strain was injected with 5% CO_2_), and ZW2 (the LE-ZW strain was injected with 10% CO_2_). The results of CK and ZW1 were compared to analyze the effects of mutagenesis and domestication on carbon metabolism of *E. gracilis*, while the results of ZW1 and ZW2 were compared to further analyze the physiological response of the mutant strain to elevated CO_2_. The cell density, carbon sequestration rate, chlorophyll content, photochemical efficiency, amount of oxygen evolution, activity for key enzymes involved in photosynthetic carbon assimilation and transcriptomics were measured in each experimental treatment group.

### 2.4 Determination index and method

#### 2.4.1 Measurement of cell density

The initial inoculum density of algal strains was 1 × 10^5^ cells/mL. The daily cell density of *E. gracilis* from 0 to 10 days in the treatment groups and the control group were measured by blood cell counting plate counting method.

Cell density = number of cells /V1.

In the formula: V1 is the volume of the blood count plate.

#### 2.4.2 Measurement of carbon sequestration rate

Each day, a total of 12 mL of algae liquid was extracted and filtered for ten consecutive days. Subsequently, the filter paper was dried, weighed before and after drying, and used to calculate the fixed CO_2_ rate (Tamburic et al., 2018).

P = (M_2_-M_1_)/(t_2_-t_1_)


$$C=\frac{P\times x}{M_{c}}\times M_{{\mathrm{CO}}_{2}}$$

In the formula: M_1_ and M_2_ represent the dry weight of algal cells (mg/L) at times t1 and t2, respectively. P denotes the driving force for biomass growth, while x represents the proportion of carbon elements in algal biomass (carbon content is calculated based on the average molecular formula of algal biomass CO0.48H1.83 N0.11P0.01). MC signifies the relative molecular weight of carbon element 12, whereas 44 stands for the relative molecular weight of CO_2_.

#### 2.4.3 Measurement of chlorophyll content

The contents of chlorophyll a (Chla), chlorophyll b (Chlb), and carotenoid (Car) in cells were determined by acetone extraction. The algal liquid was shaken well, and 8 mL of algal liquid was placed in a centrifuge tube, centrifuged at 5000 r/min for 10 min, the supernatant was removed and 5 mL of 80% acetone was added, and then crushed by ultrasonic wave for 30 min. The extracted liquid was stored in a refrigerator at 4°C for 24 h, protected from light. *E. gracilis* cells were subjected to ultrasonic extraction with acetone (40 kHz; 30 min), followed by storage at low temperature4°C for 24 h, and the absorbance values were measured at 480 nm, 510 nm, 630 nm, 664 nm, and 750 nm by spectrophotometer (Lichtenthaler and Wellburn, 1983; Sharma et al., 2020). The calculation formulas are as follows:


$$C_{\mathrm{Chla}}=\mathrm{12}\mathrm{.21}\times \left(A_{\mathrm{663}}-A_{\mathrm{750}}\right)-2\mathrm{.81}\times \left(A_{\mathrm{646}}-A_{\mathrm{750}}\right)$$

$$C_{\mathrm{Chlb}}=\mathrm{20}\mathrm{.13}\times \left(A_{\mathrm{646}}-A_{\mathrm{750}}\right)-5\mathrm{.03}\times \left(A_{\mathrm{663}}-A_{\mathrm{750}}\right)$$

$$C_{\mathrm{Car}}=7\mathrm{.6}\times \left[\left(A_{\mathrm{480}}-3\times A_{\mathrm{750}}\right)-1\mathrm{.49}\times \left(A_{\mathrm{510}}-2\times A_{\mathrm{750}}\right)\right]$$

Where, C_Chla_: chlorophyll a concentration, unit mg·L^−1^; C_Chlb_: chlorophyll b concentration, unit mg·L^−1^; C_Car_: Carotenoid concentration, unit mg·L^−1^; A_663_:663 nm absorbance; A_750_: absorbance at 750 nm; A_646_:646 nm absorbance; A_480_: absorbance at 480 nm; A_510_: Optical absorption at 510 nm.

#### 2.4.4 Measurement of photochemical efficiency

In this study, the maximum photochemical efficiency (Fv/Fm) value was selected to assess photosynthetic efficiency, and the Fv/Fm values of each treatment group were measured up to the 10th day. The Fv/Fm values were quantified using Yaxin Chlorophyll Fluorescence Analyzer (YAXIN-1161). The samples were kept in darkness for 30 min before determining Fv/Fm (Markou et al., 2016).

#### 2.4.5 Measurement of photosynthesis oxygen evolution

The dissolved oxygen (DO) content in the solution was regularly measured throughout the culture of the samples to quantify the amount of oxygen evolution. The DO values were determined using YSIPro (Lang et al., 2017).

#### 2.4.6 Measurement of activity for key enzymes involved in photosynthetic carbon assimilation

In order to further study the photosynthetic activity of LE-ZW, the enzyme activities of Ribulose-1,5-bisphosphate carboxylase/ oxygenase (RuBisCO), Fructose-1,6-diphosphatase (FBPase), Phosphoenolpyruvate carboxylase (PEPC) and Citrate synthase (CS) were determined on day 10 of cultivation, as these enzymes are key in the process of photosynthetic carbon assimilation.

The activity of RuBisCO is mainly referred to the method proposed by Stec (2012) and Rasmussen et al. (2016). The change in absorbance at 340 nm can be used to calculate the oxidation rate of nicotinamide adenine dinucleotide (NADH), which can reflect RuBisCO activity.

The activity of FBP is mainly followed the method proposed by Tamoi et al. (1998). The change in absorbance at 340 nm was monitored using a UV spectrophotometer for a duration of 2 min to assess the enzyme activity. One unit of FBP enzyme activity was defined as the quantity of enzyme that catalyzes the conversion of 1 μmol Fructose-1,6-bisphosphate per minute at 25°C.

The PEPC and CS activities were determined using spectrophotometry with the PEPC and CS assay kit. The change in absorbance at 340 nm was monitored using a UV spectrophotometer for a duration of 5 min to assess the PEPC enzyme activity. One unit of PEPC enzyme activity was defined as the quantity of enzyme that catalyzes the conversion of 1 nmol NADH per minute at 30°C. The activity of CS was determined by DTNB method, and the absorbance was measured at 412 nm for 2 min. One unit of CS enzyme activity was defined as the catalytic production of 1 nmol TNB per minute at 25°C.

#### 2.4.7 Real-time quantitative PCR (qRT-PCR)

To investigate the expression levels of photosynthetic-related proteins in LE-ZW and validate the transcriptomic findings, *psbQ*, *psbY*, *petD* and *psaD* were selected for qRT-PCR analysis. Primer Premier 5.0 software was used to design qRT-PCR-specific primers (Supplementary Table S1).

### 2.5 Transcriptome sequencing

#### 2.5.1 Library construction and sequencing

Each group had three replicates and a total of 9 samples were cultured until the 10th day. The algal solution was taken separately, centrifuged at a speed of 8,000 rpm/min for 5 min, and then the supernatant was discarded to obtain 1 × 10^8^ algal cells, which were frozen with liquid nitrogen and sent to the sequencing company. Total RNA was treated by mRNA enrichment or rRNA removal. Magnetic beads with OligodT were used to enrich mRNA with polyA tail, the rRNA was hybridized with DNA probes, the DNA/RNA hybridization strand was selectively digested with RNaseH, and the DNA probe was digested with DNaseI. The required RNA was obtained after purification. The obtained RNA was segmented by interrupting buffer, and then reverse-transcribed by random N6 primers, and then synthesized cDNA double strand to form double-stranded DNA. The end of the synthetic double-stranded DNA was patched and phosphorylated at the 5 ‘end, the 3’ end formed a sticky end with a protruding “A,” and then a bubbling connector with a protruding “T” at the 3 ‘end was connected; the connected products were amplified by PCR using specific primers. The PCR products were thermally denatured into a single strand, and then the single strand DNA was cycled with a bridge primer to obtain a single strand circular DNA library.

#### 2.5.2 Transcriptome assembly and functional notes

The transcriptome raw data was filtered using SOAP nuke, a filtering software developed by sequencing companies. The resulting clean reads were then assembled from scratch to obtain the UniGene database. BUSCO software was then used to evaluate the accuracy and completeness of the stitching results. After the transcript was obtained by Trinity concatenation, further functional annotation of the transcript was carried out. In order to obtain complete and comprehensive gene function information, the assembled UniGene was annotated with seven functional databases (KEGG, GO, NR, NT, SwissProt, Pfam, and KOG).

#### 2.5.3 Gene expression and differential expression gene analysis

The transcriptome obtained by Trinity concatenation was used as the reference sequence, and the Clean reads of each sample were compared to the reference sequence using the bowtie2 program of RSEM software. The gene expression was calculated by Fragments Per Kilobase of the exon model per Million mapped reads (FPKM). There are significant differences in the expression levels of some genes under different conditions, and these genes are called Differentially Expressed Genes (DEGs).

### 2.6 Data analysis

All experiments in this study were repeated in triplicates, and expressed in the form of mean ± standard deviation. The data were analyzed with GraphPad Pirsm software, and the significance of differences between groups was analyzed by ANOVA, with results considered to be significant at *p* < 0.05.

## 3 Results

### 3.1 The acquisition of a high-efficient carbon sequestration mutant of *Euglena gracilis*

**Figure 1:**
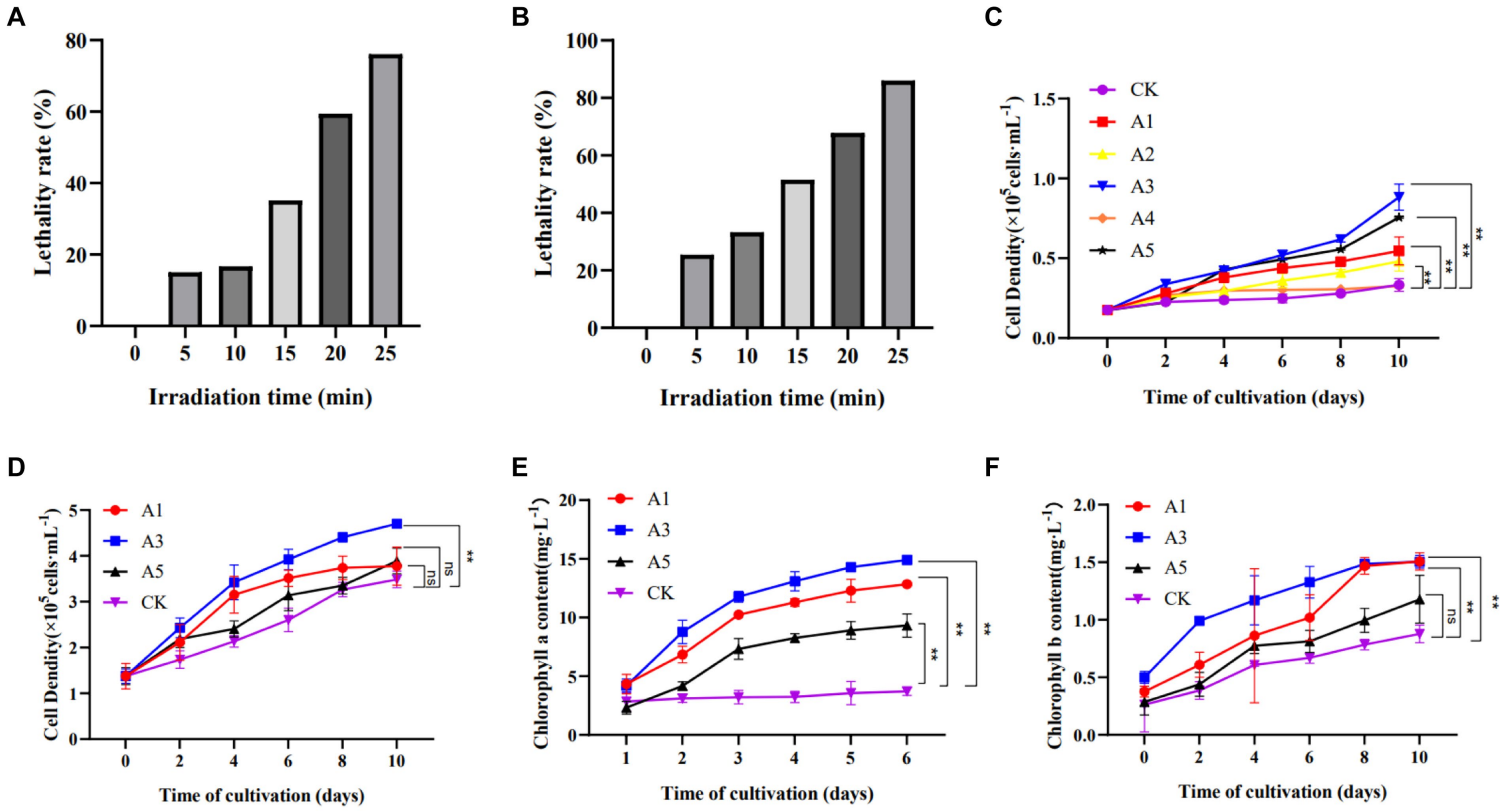
The screening of *E.gracilis* mutant strains. **(A)** The LR of UV mutagenesis is 15 cm away from UV lamp. **(B)** The LR of UV mutagenesis is 25 cm away from UV lamp. **(C)** Cell density after UV mutagenesis. **(D)** Cell density of a UV-mutagenized strain acclimated to high carbon concentration. **(E)** Chlorophyll a content of a UV-mutagenized strain acclimated to high carbon concentration. **(F)** Chlorophyll b content of a UV-mutagenized strain acclimated to high carbon concentration; The data are presented in the form of mean ± SE (*n* = 3). * and ** indicate values that differ significantly from controls at *p* < 0.05 and *p* < 0.01, respectively, according to two-way ANOVA.

After UV mutagenesis, single colonies exhibiting a large colony area and deep green color were selectively isolated on solid culture medium with an observed mortality rate of 80–90%. When the UV lamp is 25 cm and the irradiation time is 25 min, the LR is 86.06% (Figures 1A, B). Five mutant strains (A1, A2, A3, A4, A5) were initially screened. The monoclonal strains were subsequently transferred to liquid culture medium for cultivation over a period of 10 days. Further screening was conducted based on their cell density. As depicted in Figure 1C, the three groups of strains displaying the most rapid growth in cell density were identified as A3, A5, and A1 respectively; these values being 2.65 times, 2.27 times and 1.64 times higher than that of the CK group. Consequently, these three strains were selected for subsequent high carbon acclimation experiments.

**Figure 2:**
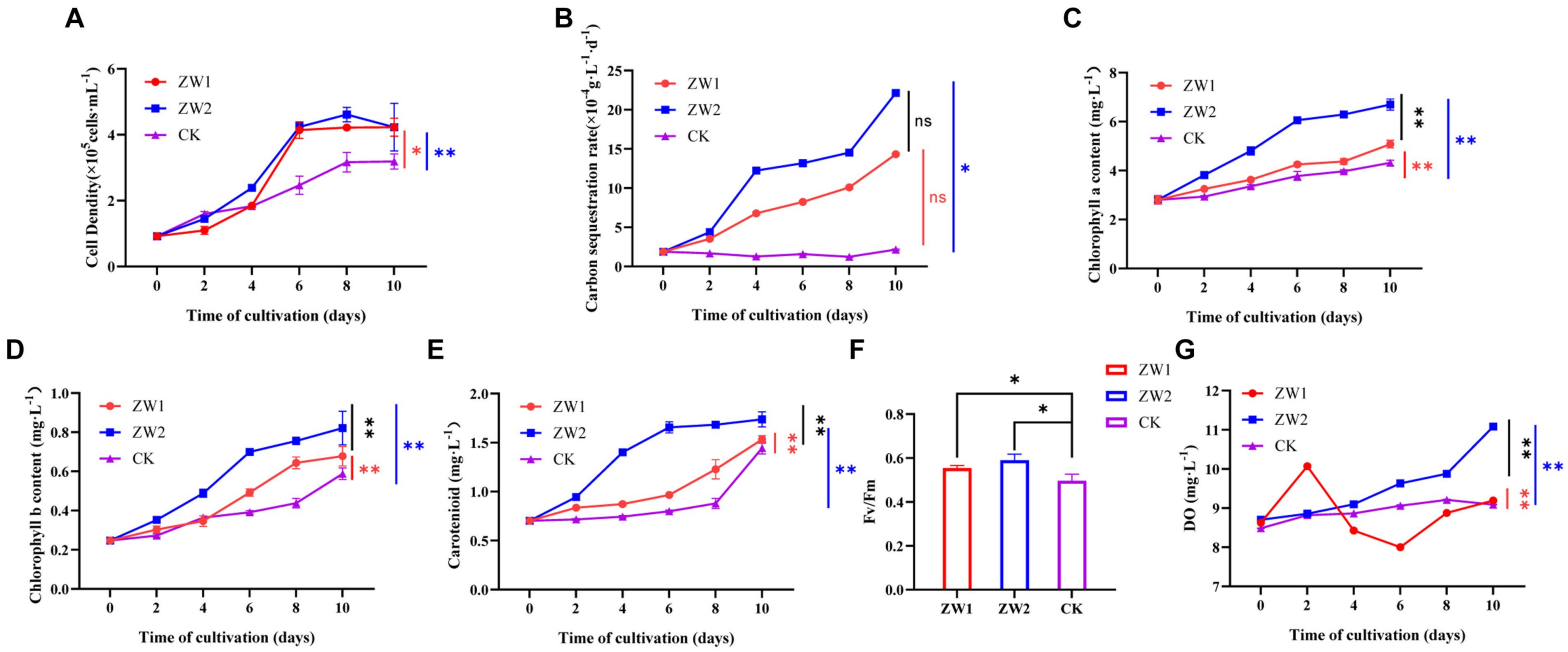
Growth and photosynthetic indices of *E. gracilis* in different treatment groups. **(A)** Cell density. **(B)** Carbon sequestration rate. **(C)** Chlorophyll a content. **(D)** Chlorophyll b content. **(E)** Carotenoid content. **(F)** Fv/Fm. **(G)** Oxygen evolution. (CK: the wild-type *E. gracilis* strain was injected with 5% CO_2_; ZW1: the LE-ZW strain was injected with 5% CO_2_; ZW2: the LE-ZW strain was injected with 10% CO_2_) The data are presented in the form of mean ± SE (*n* = 3). * and ** indicate values that differ significantly from controls at *p* < 0.05 and *p* < 0.01, respectively, according to one-way ANOVA and two-way ANOVA.

After initial screening, UV-induced mutant strains were obtained in groups A1, A3, and A5. Following high carbon acclimation, their growth was compared to the wild-type strain used as a control. During a 10-day cultivation period, cell density and photosynthetic pigment content of the algae strains were measured. As depicted in Figure 1D, the mutant strain in group A3 exhibited significantly better growth than that of the wild type (*p* < 0.05), reaching 1.35 times that of CK by the end of cultivation. The next best performing strain was group A5 with a growth rate 1.11 times that of CK followed by group A1 with a growth rate 1.08 times that of CK. Photosynthetic pigment content analysis could be employed as an important tool for screening high-efficiency carbon-fixing species. The content of chlorophyll a and chlorophyll b in group A3 was significantly higher than that of the wild type (*p* < 0.01), as depicted in Figures 1E,F. Consequently, the A3 strain, which exhibiting superior growth and photosynthetic pigment contents, were selected for subsequent experimental analysis and designated as LE-ZW.

### 3.2 Effects of UV mutagenesis and high carbon acclimation on the growth and photosynthetic characteristics of *Euglena gracilis*

After UV mutagenesis and high carbon acclimation, the growth of *E. gracilis* showed significant differences among the treatment groups. With the extension of time, the cell density in all groups showed a trend of continuous increase and reached the cell growth plateau at 8–10 days. The cell density of the ZW1 group was as high as 4.23 × 10^5^ cells/mL on day 10. The cell growth rate of the ZW2 group was significantly higher than that of the ZW1 group during the experiment, reflecting that the target algal strain had adapted to the high-carbon environment (Figure 2A). The results demonstrated that the cell density of LE-ZW was 1.33 times that of the wild type after a 10-day culture period at an optimal CO_2_ concentration of 5%. Furthermore, under a 10% CO_2_ environment for the same duration, the cell density of LE-ZW increased to 1.10 times that observed under 5% CO_2_ conditions.

The carbon sequestration rates of *E. gracilis* in the treatment groups also exhibited significant differences (Figure 2B). With the increase of time, the carbon sequestration rate of cells in the ZW1 and ZW2 groups showed a remarkable increase trend, while that in the CK group was not obvious. Under identical 5% CO_2_ concentration, the carbon sequestration rate of the algal cells after UV mutation and high carbon acclimation reached as high as 14.329 × 10^−4^ g·L^−1^·d^−1^, which was 6.67 times that of the wild-type algal strain. The carbon sequestration rate of the ZW2 group reached 22.132 × 10^−4^ g·L^−1^·d^−1^ under high carbon environment (10% CO_2_), which was 1.54 times that of the ZW1 group under low carbon environment (5% CO_2_), indicating the adaptability of LE-ZW to high CO_2_ concentration environment.

There were also significant differences in photosynthetic pigment content among experimental groups. The contents of chlorophyll a, chlorophyll b, and carotenoid in each group were basically consistent with the change trend of cell density and showed an increasing trend with the extension of culture time. By the end of the experiment, chlorophyll a content in the ZW2 group was 6.697 × 10^5^ mg·L^−1^, which was significantly higher than that in the ZW1 group (5.079 × 10^5^ mg·L^−1^) and the CK group (4.314 × 10^5^ mg·L^−1^) (Figure 2C). On the 10th day of culture, Chlorophyll b content in the ZW2 group was 1.21 times that in the ZW1 group, up to 0.352 × 10^5^ mg·L^−1^, while that in the ZW1 group was 1.15 times that in the CK group, up to 0.303 × 10^5^ mg·L^−1^ (Figure 2D). Carotenoid content in the ZW2 group showed an increasing trend in the first 6 days of growth, reaching 1.66 × 10^5^ mg·L^−1^ on the 6th day, and then remained relatively stable until the end of the experiment. However, carotenoid content in the ZW1 group and the CK group increased slowly in the first 8 days of growth, and then increased rapidly, reaching 1.53 × 10^5^ mg·L^−1^ in the ZW1 group and 1.44 × 10^5^ mg·L^−1^ in the CK group on the 10th day (Figure 2E). Measurements of the Fv/Fm values were conducted on algae strains cultivated up to the 10th day, the Fv/Fm values of ZW2 and ZW1 were significantly higher than those of the control group, being 1.18 times and 1.11 times that of CK (Figure 2F). The oxygen evolution levels of each experimental group were measured during culture. The results showed that the DO value of ZW2 was the highest (11.08 mg·L^−1^) on the 10th day, which was significantly higher than the other two groups (*p* < 0.01). The oxygen evolution in CK group increased slightly during culture, and the trend was steady. However, ZW1 showed the highest oxygen evolution at 10.07 mg·L^−1^ on the second day of culture, and then the oxygen evolution decreased and was similar to CK on the 10th day of culture (Figure 2G).

#### 3.2.1 Enzyme activity of key enzymes related to photosynthetic carbon metabolism

**Figure 3:**
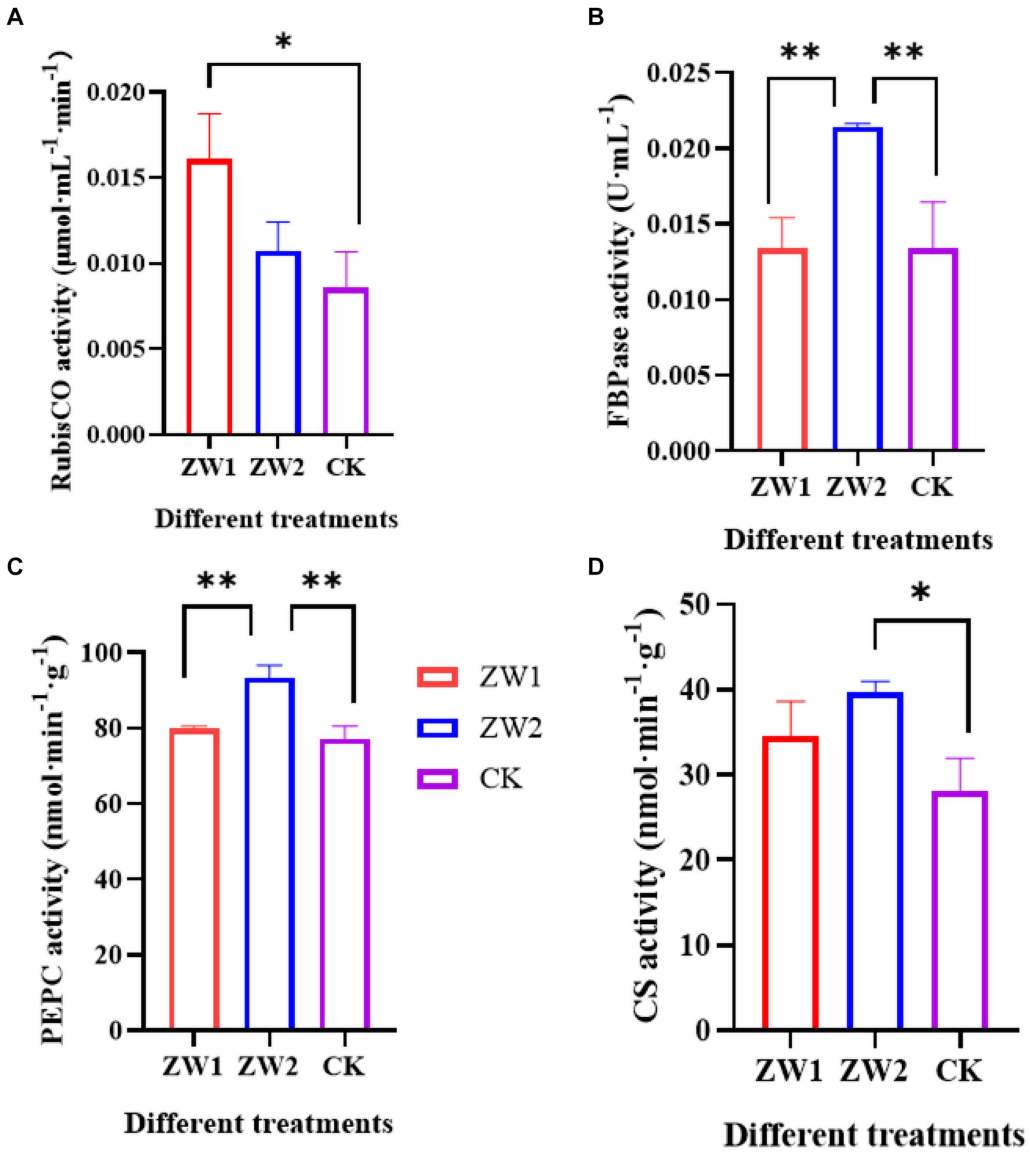
Activity of key enzymes for photosynthetic carbon assimilation in samples cultured on the 10th day. **(A)** RubisCO activity. **(B)** FBPase activity. **(C)** PEPC activity; **(D)** CS activity. The data are presented in the form of mean ± SE (*n* = 3). * and ** indicate values that differ significantly from controls at *p* < 0.05 and *p* < 0.01, respectively, according to one-way ANOVA.

The activities of several key enzymes associated with the photosynthetic carbon sequestration pathway were measured in each experimental group on the 10th day of cultivation. Rubisco acts as the key enzyme during photosynthesis and the rate limiting enzyme during Calvin cycle by catalyzing the first step in the reduction of CO_2_ in photosynthetic organisms. As shown in Figure 3A, ZW1 exhibits the highest Rubisco enzyme activity at 0.016 μmol·mL^−1^·min^−1^, followed by the ZW2 group at 0.0107 μmol·mL^−1^·min^−1^. FBP is a pivotal enzyme in the sugar biosynthesis pathway and carbon fixation during photosynthesis. As depicted in Figure 3B, ZW2 demonstrates the highest FBP enzyme activity at 0.0214 U·mL^−1^, while ZW1 shows similar enzyme activity levels to CK. PEPC catalyzes the irreversible reaction between phosphoenolpyruvate and carbon dioxide. At the conclusion of the culture period in Figure 3C, ZW2 exhibited the highest PEPC enzyme activity at 93.33 nmol·min^−1^·g^−1^, whereas ZW1 displayed PEPC enzyme activity comparable to CK. CS functions as the primary rate-limiting enzyme in the tricarboxylic acid cycle, facilitating the conversion of acetyl-CoA into citryl-CoA and oxaloacetate, as illustrated in Figure 3D. The ZW2 group exhibited the highest CS activity at 39.66 nmol·min^−1^·g^−1^, followed by the ZW1 group at 34.52 nmol·min^−1^·g^−1^.

### 3.3 Effects of UV mutagenesis and high carbon acclimation on the transcriptome of *Euglena gracilis*

#### 3.3.1 Transcriptome data quality control analysis

**Figure 4:**
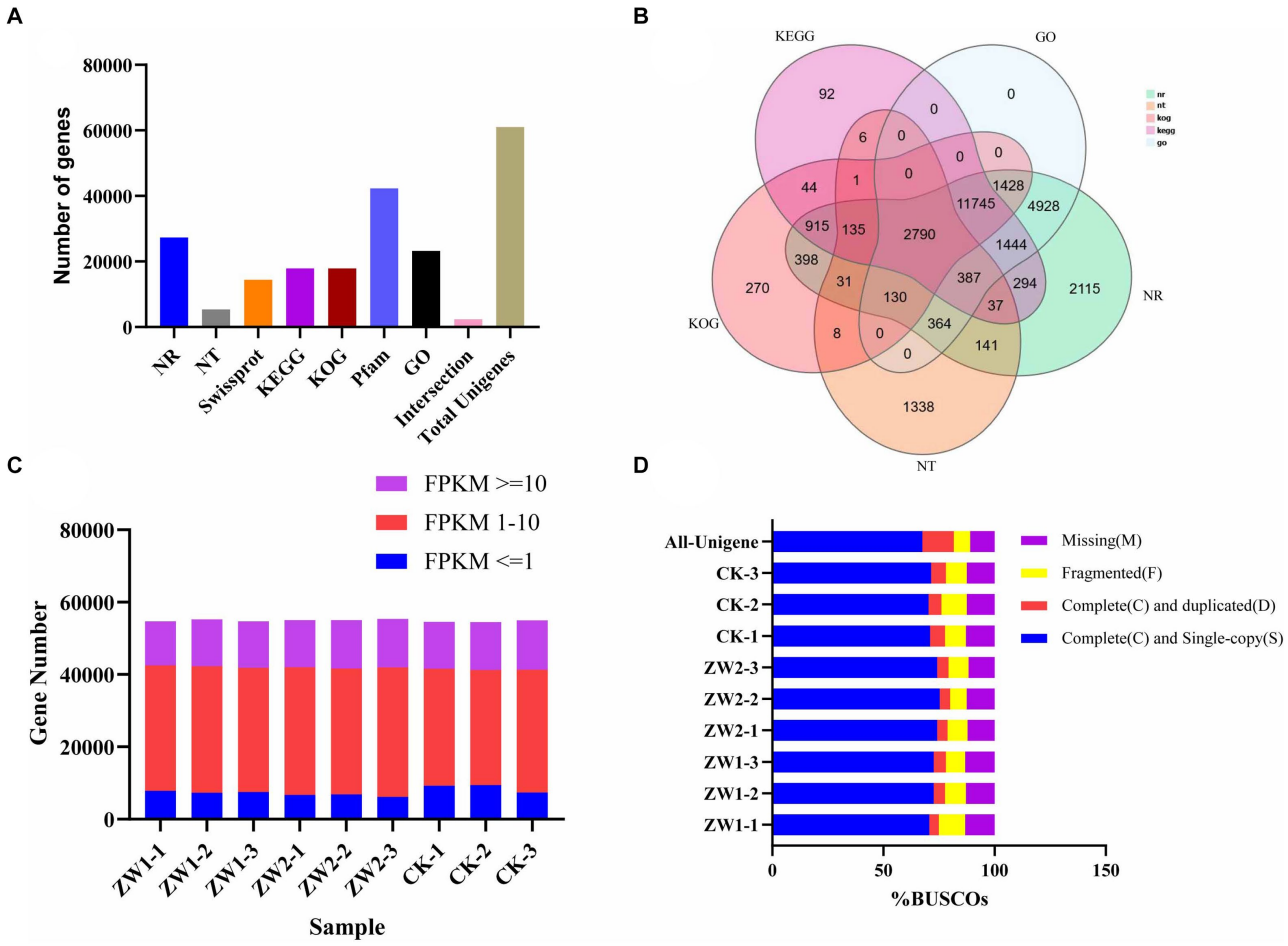
*Euglena gracilis* Quality evaluation of mRNA sequencing data. **(A)** Gene annotation success rate statistics. **(B)** Venn map of differential genes. **(C)** FPKM density distribution of gene expression in samples. **(D)** BUSCO evaluation results of spliced transcripts.

Seven databases were used to annotate gene functions of the splicing transcripts, and a total of 61,062 UniGene annotation information was obtained (Figure 4A). Total RNA sequencing and assembly were performed on 9 samples from 3 experimental groups. After filtering and screening, about 42,533,333, 43,080,000, and 43,203,333 Clean reads were obtained from ZW1, ZW2, and CK, respectively (Figure 4B). According to the preliminary assessment of transcriptome sequencing quality of the wild-type strains and the mutant strains under different CO_2_ aeration concentrations, the data were reliable for subsequent analysis (Figure 4C). Evaluation of assembled transcripts against BUSCO software can demonstrate the integrity of their assembly (Figure 4D).

#### 3.3.2 Functional annotation of differentially expressed genes

**Figure 5:**
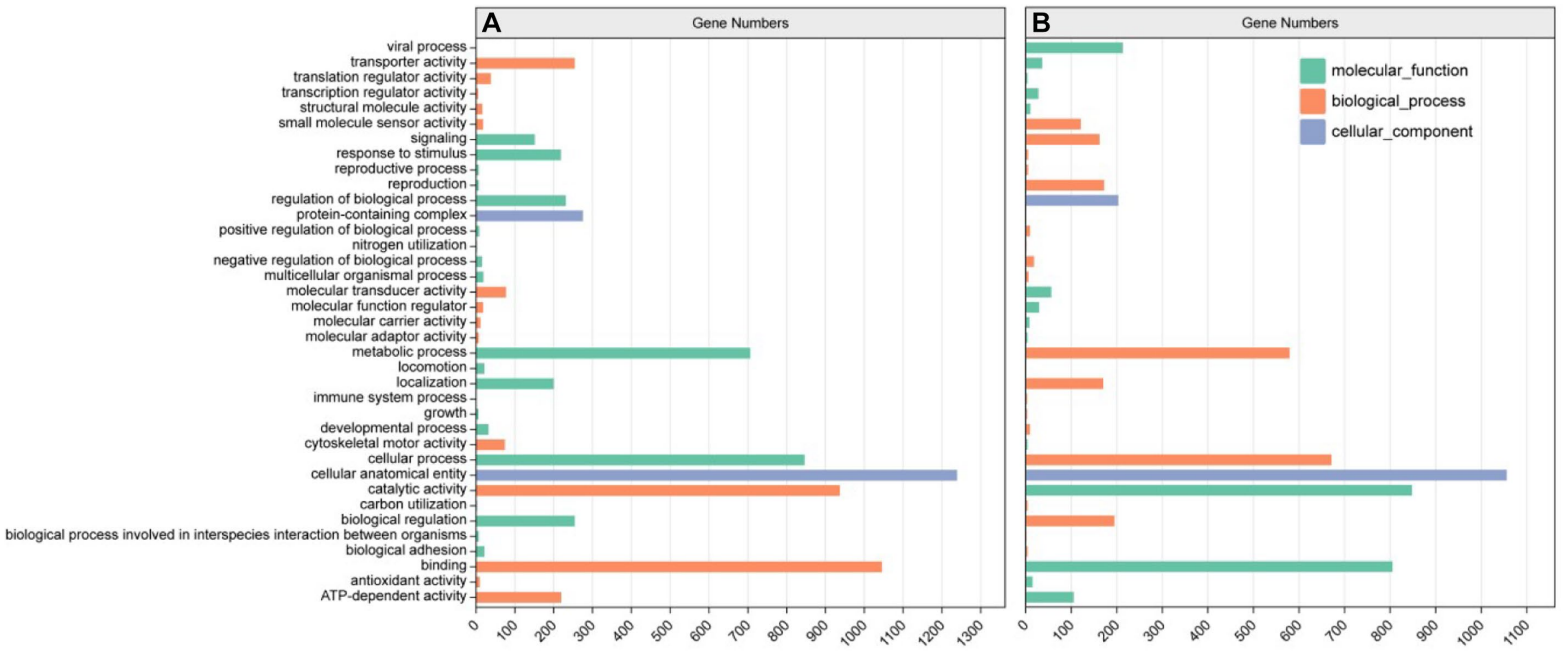
GO annotation classification statistics. **(A)** ZW1 vs. CK. **(B)** ZW1 vs. ZW2.

Most of the genes of *E. gracilis* were enriched in 53,543 UniGene notes for biological processes (BP), 47,933 UniGene notes for molecular functions (MF), and 25,721UniGene notes for cell components (CC) (Figure 5). According to the result of GO secondary classification, metabolic process annotation reached 12896UniGene, biological process regulation annotation reached 4,558 UniGene, signaling pathway annotation reached 3378UniGene, antioxidant activity annotation reached 233 UniGene and carbon utilization annotation reached 15 UniGene, etc.

**Figure 6:**
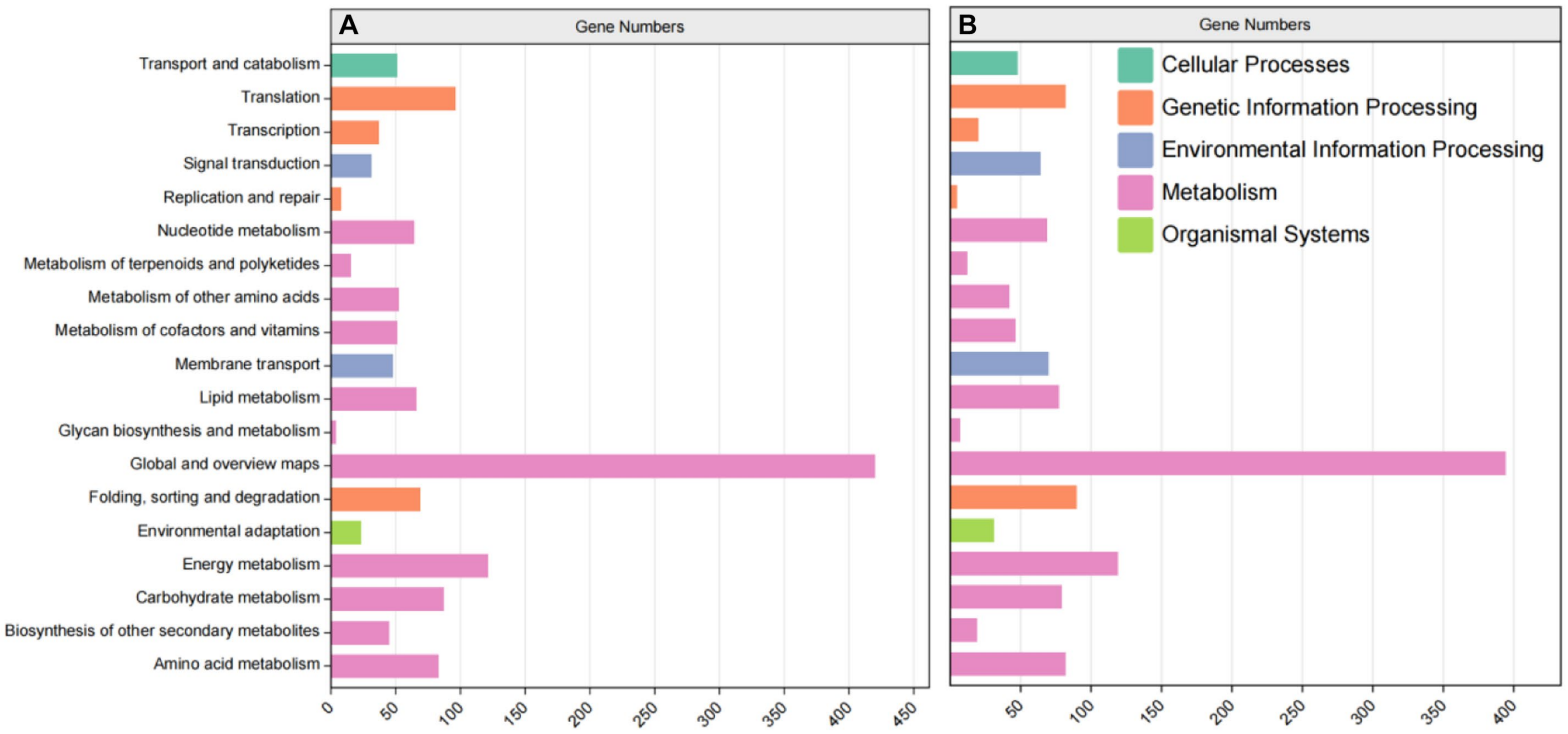
KEGG metabolic pathway classification statistics. **(A)** ZW1 vs. CK. **(B)** ZW1 vs. ZW2.

KEGG metabolism pathway can be divided into 5 categories according to genes involved. According to the results, the categories with more annotations included 11,311 UniGene annotations for metabolism, 2,984 UniGene annotations for genetic information processing, 1,145 UniGene annotations for environmental information processing, 728 UniGene annotations for cellular processes and 447 UniGene annotations for organic systems (Figure 6). In order to deeply compare the mechanism of carbon metabolism among different experimental treatment groups, the metabolic process was analyzed. In these genetic sequences, there were 985 UniGene gene annotations for carbohydrate metabolism, 1920 UniGene gene annotations for nucleotide metabolism, and 763 UniGene gene annotations for energy metabolism, etc.

#### 3.3.3 Analysis of differentially expressed genes

**Figure 7:**
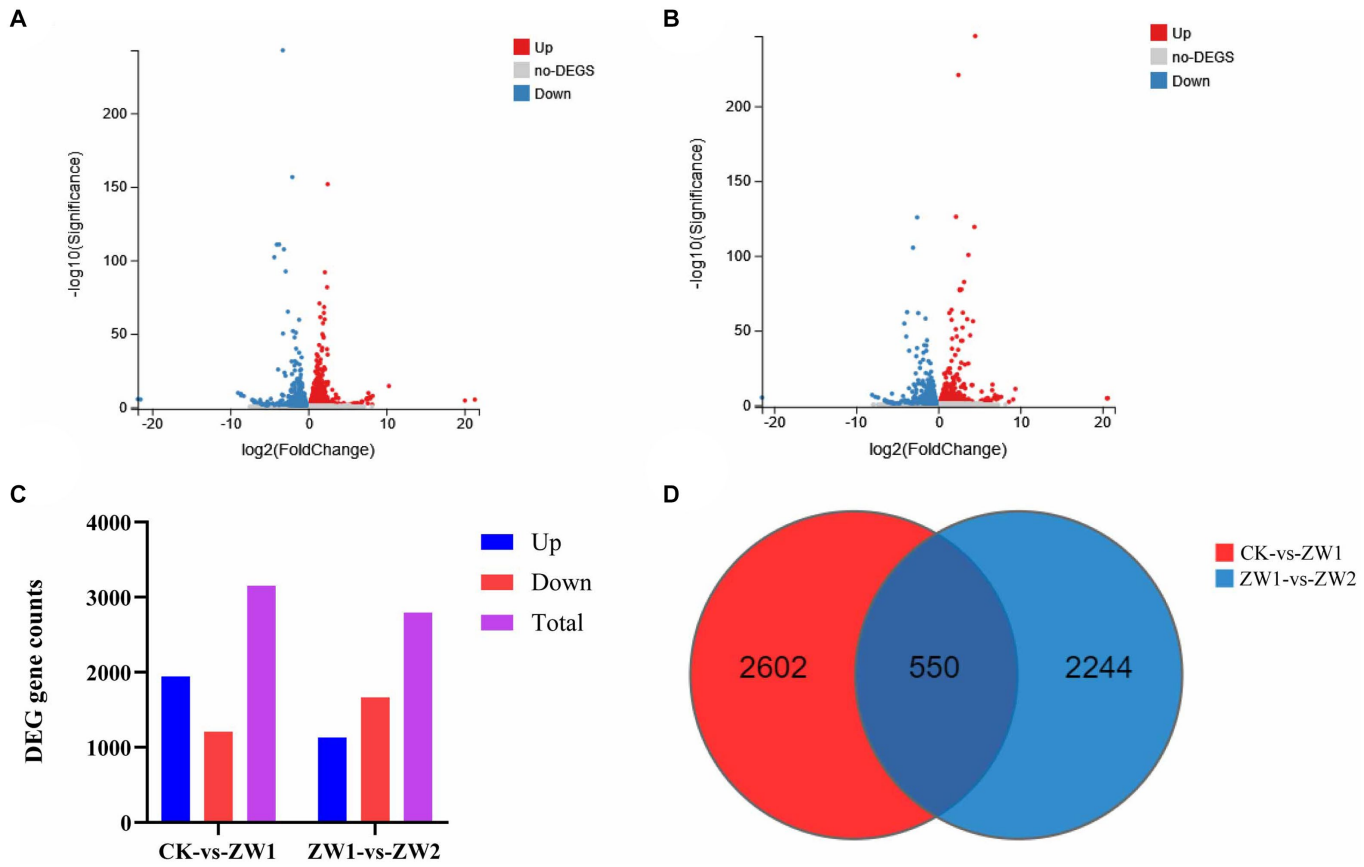
Transcriptional responses of *E. gracilis* to high CO_2_ stress. **(A)** DEGs volcano map analysis of group ZW1 vs. group CK. **(B)** DEGs volcano map analysis of group ZW1 vs. group ZW2. **(C)** The number of up-regulated and down-regulated DEGs between different groups. **(D)** DEGs Venn diagram between different groups.

Pairwise transcriptome analyses were performed to identify DEGs, and the expression levels of DEGs in the three experimental treatment groups were compared. Compared with the CK group, there were 3,152 differential genes in the ZW1 group, including 1945 up-regulated genes and 1,207 down-regulated genes. Compared with the ZW1 group, there were 2,794 differential genes in the ZW2 group, including 1,130 differential genes up-regulated genes and 1,664 differential genes down-regulated genes (Figure 7).

#### 3.3.4 Enrichment analysis of differentially expressed genes

**Figure 8:**
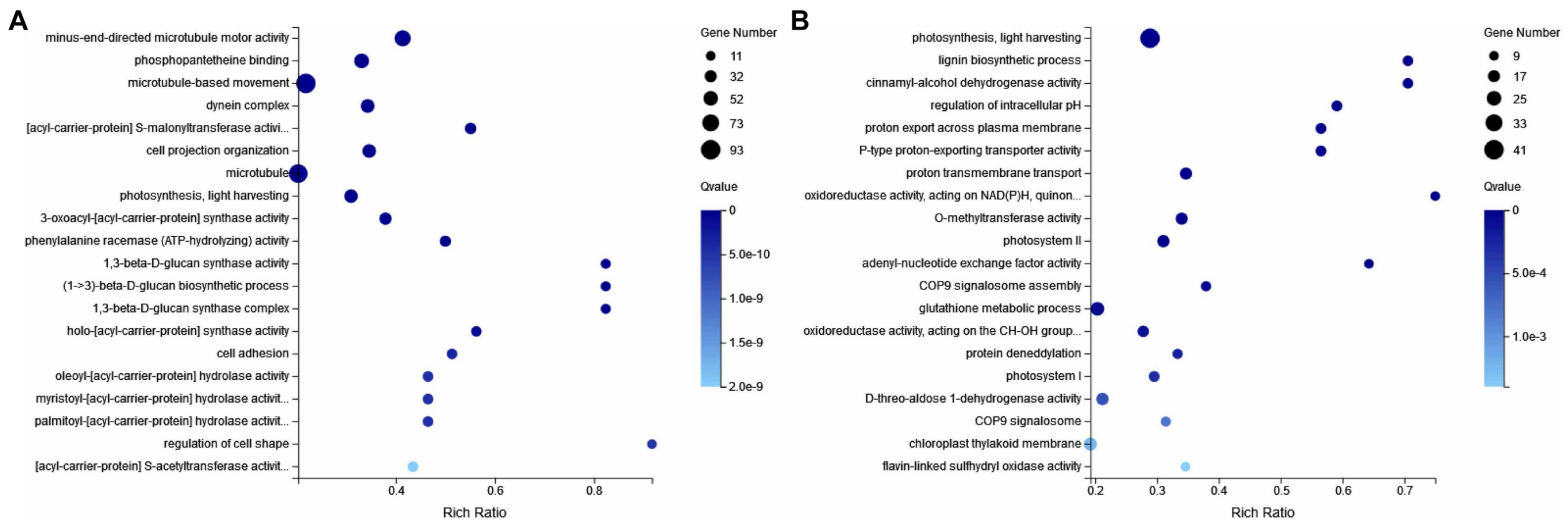
GO enrichment of differential genes. **(A)** CK vs. ZW1. **(B)** ZW1 vs. ZW2.

To further elucidate the response of *E. gracilis* under high CO_2_ conditions after UV mutagenesis and high carbon acclimation, DEGs between the CK group and the ZW1 group, and between the ZW1 group and the ZW2 group were subjected to GO analysis. The GO enrichment analysis between the CK group and the ZW1 group showed that 1,098 DEGs were enriched into three categories: BP, CC, and MF. The detected DEGs were annotated as 147 cell components, 446 biological processes, and 504 molecular functions. Within the category of BP, translation (GO:0006412), peptide biosynthesis process (GO:0043043), cellular process (GO:0060285), metabolic process (GO:0006075) and developmental process (GO:0008360) were prominent. Within the category of CC, dynein complex (GO:0030286), microtubule (GO:0005874), and axonemal dynein complex (GO:0005858), were significantly enriched. Within the category of MF, catalytic activity (GO:0004318), transporter activity (GO:1904680), and ATP-dependent activity (GO:0042626) were significantly enriched (Figure 8A). The GO enrichment analysis of the differential genes in ZW1 and ZW2 groups showed that 1,037 DEGs were enriched into the three GO categories. The detected DEGs were annotated as 156 cell components, 417 biological processes, and 463 molecular functions. The significant enrichment of chloroplast (GO:0009507,65) related genes in BP. There was a significant enrichment of genes related to photosynthesis (GO:0009765,41) and carbon utilization (GO:0015976,4) in CC. The significant enrichment of transmembrane transporter activity (GO:0022857,59), and intracellular signal transduction (GO:0035556,95) related genes in MF (Figure 8B).

**Figure 9:**
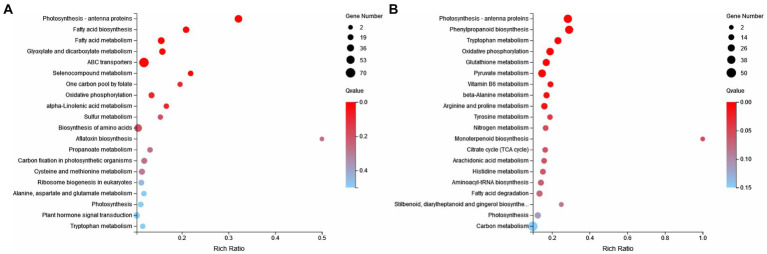
KEGG enrichment of differential genes. **(A)** CK vs. ZW1. **(B)** ZW1 vs. ZW2.

KEGG enrichment analysis was carried out. Compared with the CK group, metabolic pathways in the ZW1 group were mainly enriched at Citrate cycle (ko00020), ABC transporters (ko02010), Fatty acid metabolism (ko01212), Carbon fixation in photosynthetic organisms (ko00710) and Oxidative phosphorylation (ko00190) (Figure 9A). Compared with the ZW1 group, metabolic pathways in the ZW2 group were mainly enriched at Carbon metabolism (ko01200), Photosynthesis-antenna proteins (ko00196), Biosynthesis of amino acids (ko01230), and Carbon fixation in photosynthetic organisms (ko00710) (Figure 9B).

#### 3.3.5 qRT-PCR analysis of differentially expressed genes in LE-ZW

**Figure 10:**
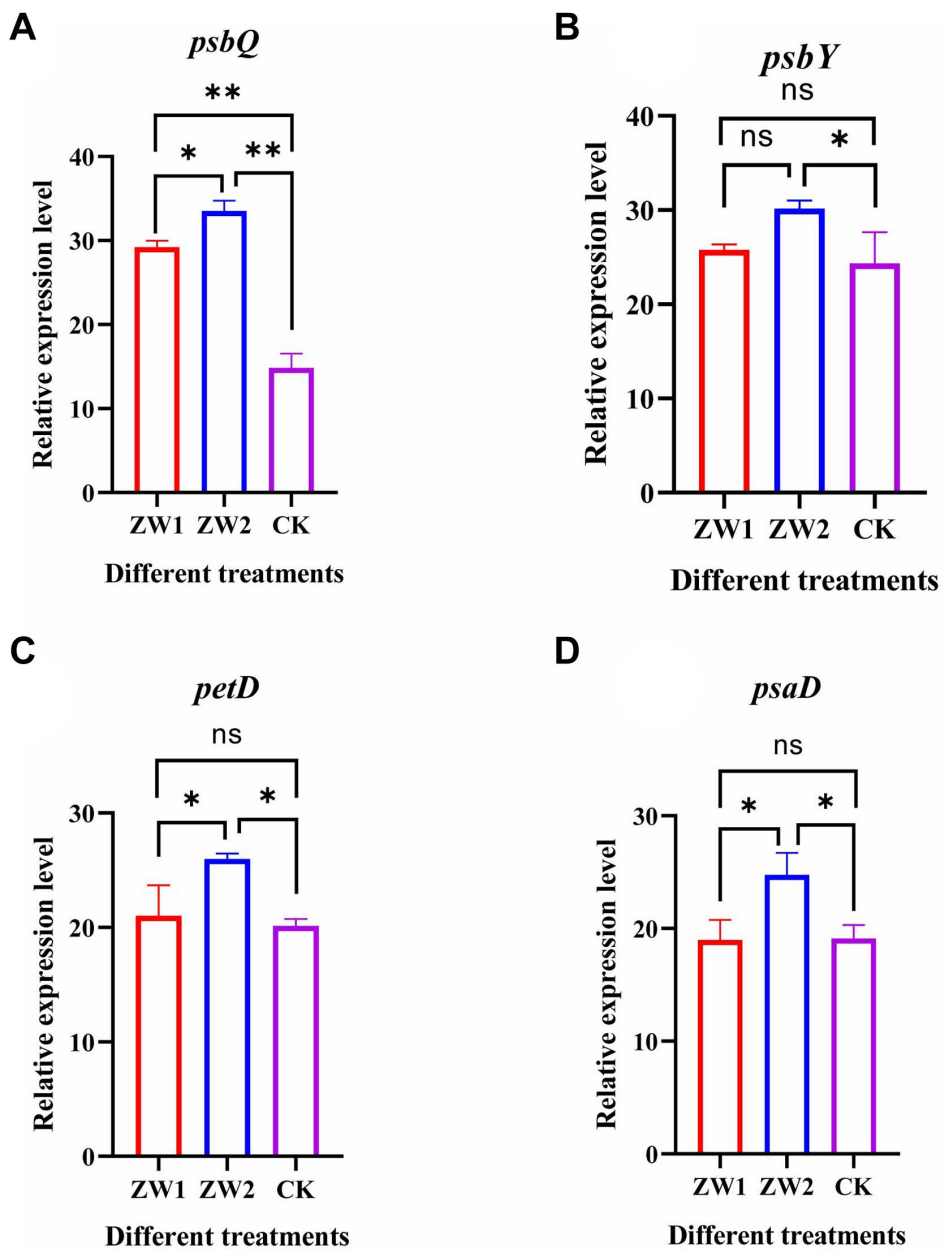
Expression levels of genes related to photosynthesis in LE-ZW. **(A)** *psbQ*; **(B)** *psbY*; **(C)** *petD*; **(D)** *psaD*. The data are presented in the form of mean ± SE (*n* = 3). * and ** indicate values that differ significantly from controls at *p* < 0.05 and *p* < 0.01, respectively, according to one-way ANOVA.

The expression levels of the photosynthesis protein-related genes *psbQ*, *psbY*, *petD* and *psaD* were quantified in mutant LE-ZW under varying CO_2_ concentrations. The results revealed significant upregulation of the *psbQ* gene in ZW1 compared to CK, and all genes (*psbQ*, *psbY*, *petD* and *psaD*) in ZW2 exhibited significant upregulation consistent with the transcriptomics data (see Figure 10)

## 4 Discussions

### 4.1 Effects of UV mutagenesis and high-carbon acclimation on photosynthesis of *Euglena gracilis*

Photosynthesis is closely related to the growth of *E. gracilis*. Photosynthetic pigments are important components of algae to capture light and transfer light quantum, and the change of photosynthetic pigment content can be used as an index to detect the difference of photosynthetic efficiency. The photosynthetic pigments of *E. gracilis* mainly include chlorophyll and carotenoids (Shunqin et al., 2021). Chlorophyll a plays a major role in the light-trapping system of algal cells, and chlorophyll b can effectively capture blue-violet light and carry out its photosynthesis (Lu et al., 2020). The light-trapping pigments represented by chlorophyll a and chlorophyll b can effectively transfer the absorbed light energy to the relevant reaction center and convert it into chemical energy. In addition to the light-trapping function, carotenoid also participates in the regulation of photoprotection, which can absorb residual energy, to avoid the injury of the cell membrane (Hou et al., 2016). Under the same CO_2_ concentration, the contents of chlorophyll a, chlorophyll b, and carotenoids increased significantly after UV mutagenesis combined with high carbon acclimation, and the ability of algal cells to capture light was enhanced, thus, the anabolism of algal cells was improved, and the photosynthetic efficiency of algal cells was further promoted. The Fv/Fm parameter serves as a reliable indicator of photosynthetic efficiency, with higher values indicating enhanced absorption and utilization of light energy by the organism. Additionally, photosynthetic oxygen evolution is a crucial and direct metric for assessing plant photosynthetic efficiency. Based on the Fv/Fm values and photosynthetic oxygen evolution data obtained from this study, it was confirmed that LE-ZW exhibited higher photosynthetic efficiency compared to the wild type.

Photosynthesis is also an important nutrient conversion pathway in plants (Ma and Wang, 2021). KEGG enrichment analysis in this study showed that the mutant strain (LE-ZW) obtained after UV mutation and high carbon acclimation treatment significantly increased the expression of genes related to photosynthesis and glucose metabolism compared with the wild-type algae under the same CO_2_ concentration. The significantly upregulated genes in LE-ZW are mainly related to the photosystem membrane protein complex, photosynthetic electron transport chain, antenna protein, carbon fixation and glycolysis. PSI is mainly involved in the non-cyclic electron transport process in photosynthesis to produce ATP and NADPH. PSI is not directly involved in the splitting of water and the production of oxygen, which belongs to the auxiliary electron transport system, and its role in the photoreaction is to transfer the reprocessed electrons to NADP^+^ for reduction to NADPH (Ho et al., 2011). The up-regulated expression of psbA and psbB genes, encoding the PSI protein complex, enhances electron transport efficiency around PSI, thereby augmenting the rate of photosynthetic carbon sequestration. In this process, the PSI protein complex can improve the supply of ATP and the REDOX state of the thylakoid membrane matrix under stress conditions. PSII is the main reaction center of photosynthesis to produce oxygen and release the electrons and protons (Vangelis, 2018). It removes electrons from water molecules by absorbing high-energy photons and splits the water molecules into oxygen and hydrogen ions, capturing the energy and converting it into the chemical energy of ATP (Roberto and Luca, 2021). The expression of PSII protein complex coding genes psbM, psbO, psbP, psbQ and psbR were significantly up-regulated in the ZW1 treatment group. This finding suggests that LE-ZW accelerates the synthesis of ATP and NADPH_2_ compared with the wild-type algae. Increased levels of ATP and NADPH_2_ further promote the conversion of active chemical energy into stable chemical energy stored in carbohydrates. The conversion of light energy into chemical energy is carried out on the thylakoid, so the thylakoid membrane is also called the photosynthetic membrane (Minrui et al., 2015). The photosynthetic electron transport chain is mainly composed of PSI, PSII, and CyT-B6 /f complexes on the photosynthetic membrane (Xiugong et al., 2003). In the results of this study, the expression of PetF encoding the CyT-B6/f complex subunit was up-regulated in the ZW1 treatment group, it can be inferred that LE-ZW algae strain promoted the conversion of light energy to chemical energy on the one hand, and also promoted the electron transfer of photosynthesis on the other hand. The photo-trapping proteins of PS I and PS II in algae are LHC I and LHC II, respectively, encoded by the corresponding nuclear genes (Lhca and Lhcb) (Viviana et al., 2016). The LHC I and LHC II bind pigments such as chlorophyll and carotene and are inserted into the thylakoid membrane to improve the transfer speed of photosynthetic electron chains. The results showed that the expressions of lhcb2 and lhca1 genes were up-regulated in the LE-ZW alga strain, suggesting that the combination of UV mutation and high carbon acclimation activated the PSI-LHCI super-complex in alga cells, accelerated photosynthetic electron transfer, and promoted the growth and photosynthesis.

Introducing appropriate CO_2_ gas during microalgae growth enhances photosynthetic electron transport between PSII and PSI, thereby increasing the photon yield and energy for CO_2_ fixation, enhancing the photosynthetic rate of algal cells, and promoting the carbon sequestration effect of microalgae (Chen et al., 2022). At the core of photosynthesis are two major photoactive complexes PSI and PSII that channel sunlight into the electron transport chain via excited chlorophyll dimers (van Bezouwen et al., 2017). In the ZW2 group, the up-regulation of psbD expression of the gene encoding PSI protein complex indicated that LE-ZW enhances the formation and electron transfer rate of the PSII-PSI complex under 10% CO_2_ concentration, thereby establishing an efficient electron transport pathway in the photosynthetic membrane. This optimization of energy transfer pathways led to a higher carbon fixation photosynthetic efficiency. It is important to note that light energy absorption is a prerequisite for PSII function. The expression of PSII protein complex coding genes such as psbM, psbO, psbP, psbQ, psbR, psbW, psbY, psb28, and psbT, exhibited up-regulation in the ZW2-treated group. This observation suggests an enhanced light energy conversion efficiency of the mutant LE-ZW under 10% CO_2_ concentration. The Cyt B6/f complex is positioned between PSII and PSI and participates in both linear and cyclic electron transport (Cramer and Zhang, 2006). The results demonstrated an up-regulation in the expression of the petD gene, which encodes Cyt B6/f complex subunit, upon injection of 10% CO_2_ was injected. It can be inferred that compared with ZW1, the algal cells of the ZW2 group optimized and modified the energy transfer mode on the photosynthetic membrane, thereby enhancing the light energy utilization efficiency. The high expression of several photosynthesis-related protein genes *psbQ*, *psbY*, *petD* and *psaD* in the qRT-PCR results also confirmed the transcriptome results. Furthermore, this study revealed that elevated CO_2_ concentrations significantly enhance the expression of key genes involved in carbon fixation, accelerate photosynthetic electron transfer, and promote the growth and photosynthesis of the LE-ZW strain.

### 4.2 Effects of UV mutagenesis and high-carbon acclimation on the carbon fixation pathways of *Euglena gracilis*

The key enzyme cycle in Calvin’s carbon fixation process, Rubisco enzyme, plays a crucial role in catalyzing CO_2_ conversion into organic sugars (Khamis et al., 2023). The activity of this enzyme is co-regulated by several genes (Lu et al., 2023). The transcriptome results of this study showed that compared with CK, Xylulose-5P (5.1.3.1), Glyce ralde-3p (1.2.1.13), and ribo-5p (2.2.1.1) were significantly expressed in ZW1. Since the Rubisco enzyme is the rate-limiting enzyme of carbon assimilation in photosynthesis, the increase in the expression of Rubisco enzymes-related genes enhanced the carboxylation activity of Rubisco enzyme, thus improving the efficiency of CO_2_ assimilation (Hu et al., 2016). The results of enzyme activity determination in this study also confirmed that the Rubisco enzyme activity of LE-ZW was significantly higher than that of the wild type, thus validating the transcriptome results.

Some studies have shown that high concentration of CO_2_ increased the activity of Rubisco, sedoheptulose-1, 7-diphosphatase (SBP), FBP, PEPC and other enzymes (Inger, 2008; Dias, 2022; Xuehe et al., 2022). The significant expression of these key enzyme genes effectively promoted the ability of *E. gracilis* to fix CO_2_, and further improved the efficiency of photosynthesis and biomass. The high concentration of CO_2_ concurrently facilitated the augmentation of photosynthetic pigment contents and carbon sequestration rate. Among them, Rubisco is a complex structure composed of 8 large and 8 small subunits (L8S8) (Luca et al., 2022). Transcriptome analysis showed that the expression levels of the genes encoding small subunits, Glyce ralde hyde-3P (1.2.1.12) and D-Fructose 1,6P_2_ (4.1.2.13), were down-regulated in the ZW2 group, while the expression levels of large subunit genes, Ribulose-5P (5.3.1.6), glycerol-3p (4.1.1.39) and 1, 3-bisphospho glycerate (2.7.2.3), were up-regulated. The large subunits are responsible for the center of catalytic activity, while the small subunits play a unique role in regulating catalytic activity (Hayer-Hartl and Hartl, 2020). However, the specific effect of down-regulating small subunit expression on the Calvin cycle is not apparent (Andersson and Backlund, 2008). In the enzyme activity experiment, although there was no significant increase in the activity of Rubisco enzyme in ZW2, both FBP enzyme and PEPC enzyme exhibited an upward trend in their activities, thereby corroborating our transcriptomic findings. The photosynthesis of LE-ZW was enhanced under high CO_2_ concentrations while experiencing a decrease in Rubisco enzyme activity due to the “source and sink” equilibrium theory. Initially, higher concentrations of CO_2_ stimulated increased production of photosynthetic products (the source). However, as culture time progressed without a corresponding increase in storage capacity for these accumulated products (the sink), limitations arose. As a result, prolonged culture time led to feedback inhibition on photosynthetic product accumulation and subsequent reduction in Rubisco enzyme activity (Zhang, 1999).

Carbonic anhydrase (CA) also plays an important role in the carbon fixation pathway, especially in the carbon concentration mechanism. In this study, transcriptomic sequencing found that there were no significant differences in the expression of all the key genes (*Car2*, *Car1*, etc., a total of 5 key genes were screened) encoding carbonic anhydrase between groups ZW1, ZW2 and CK, indicating that CO_2_ was sufficient in the solution of each group under the condition of continuous CO_2_ injection, and carbon concentration was not necessary. It is speculated that CO_2_ directly penetrates into *E. gracilis* cells to participate in the Calvin cycle reaction mainly by osmotic pressure. This process reduces the active transport of HCO_3_^−^ and saves more energy. In the preliminary experiment, it was observed that the sample solutions of each group were acidic due to the continuous introduction of CO_2_, and the pH of each group reached about 3 during 3–4 days of culture. Researchers have discovered that the solubility of CO_2_ in fresh water ranges from 10 to 15 μmol·L^−1^, while in acidic water, especially when pH < 5.0, most inorganic carbon exists in the form of CO_2_. Thus, algae that grow under acidic pH conditions may encounter limitations in terms of inorganic carbon availability (Gross, 2000). *E. gracilis* is an acidophilic species, so it can rely on CO_2_ diffusion to provide substrate for photosynthesis in an acidic environment with sufficient CO_2_, and it grows well.

**Figure 11:**
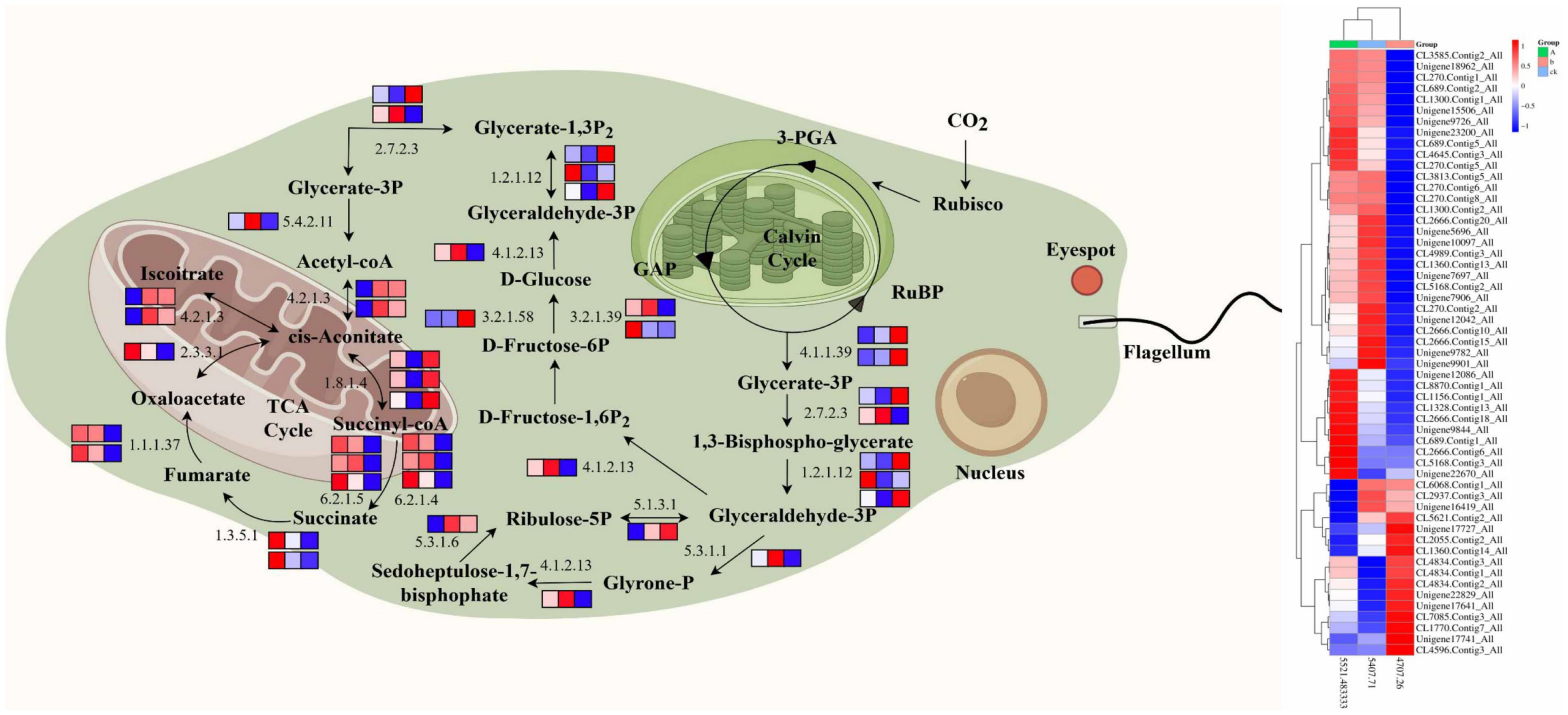
Changes in transcription levels of key enzymes in carbon metabolic pathways.

This study demonstrated that high CO_2_ concentration increased the expression and activity of key enzyme genes in the carbon sequestration pathway of LE-ZW, which was also confirmed in *Dunaliella salina* (Sheng-Yi et al., 2008). These results indicated that target algae strains possess adaptability to high concentrations of CO_2_ as well as efficient carbon sequestration capabilities (Figure 11).

### 4.3 Effects of UV mutagenesis and high-carbon acclimation on the carbon metabolic pathway of *Euglena gracilis*

Central carbon metabolism pathway is one of the important metabolic systems in organisms, which is the main pathway for converting carbon sources into energy and biomacromolecules in organisms (Lv et al., 2019). Acetyl-coA (6.2.1.1) and other genes related to central carbon metabolism pathway were also significantly expressed (Cheng et al., 2017). Although glycolysis and gluconeogenesis share most reversible enzymes, such as β-D-Fructose-6P (2.7.1.90), Glyce ralde hyde-3P (4.1.2.13), α-D-Glucose (2.7.1.1), Acetalde hycle (1.1.1.1), they employ distinct enzymes in certain crucial steps (Lv et al., 2019). Notably, Glyceraldehyde-3P is associated with glycolysis, while pyruvate carboxylase and fructose-1, 6-phosphatase are involved in gluconeogenesis among others. The gene expression of key enzymes in metabolism directly influences the velocity and directionality of the entire metabolic pathway. The transcriptomic results of this study revealed compared to the control group (CK), the expression levels of key enzyme genes encoding TCA, such as Acetyl-coA (6.2.1.1), Acetalde hyde (1.1.1.1), and Acetate (1.2.1.3), were up-regulated in the ZW1 group, indicating an enhanced metabolic rate for increased NADPH production and carbon skeleton synthesis for biosynthesis purposes. However, there was a down-regulation observed in the expression levels of Citrate (4.2.1.3) and Glyce ralde hyde-3P (4.1.2.13). This decrease could be attributed to the diversion of intermediate products from the TCA cycle towards amino acid metabolism or other pathways (Figure 11).

The decomposition products of sugar, fat, and protein are primarily metabolized into CO_2_, H_2_O, and ATP through the TCA cycle. The TCA cycle is a common pathway for the oxidation function of three major nutrients in organisms (Muñoz et al., 2021). The TCA cycle has a strict regulatory system, which is mainly regulated by three key enzymes, isocitrate dehydrogenase acts as the pivotal regulatory point, followed by α-ketoglutarate dehydrogenase complex and CS (Yamori and Shikanai, 2016). The results of enzyme activity in this study demonstrated that 10%CO_2_ enhanced the activity of CS, while simultaneously upregulating the expression of crucial genes involved in glycolysis, TCA cycle, and photorespiration activity, thus promoting carbon sequestration and biomass synthesis in LE-ZW. In transcriptome analysis, compared with the ZW1 group, up-regulation was observed in Ethanol (1.1.1.2) and Glycerate-3P (5.4.2.11) genes encoding key enzymes within the TCA pathway in the ZW2 group; however, down-regulation was observed in Lipomaide-E (1.8.1.4) expression gene. A high concentration of CO_2_ facilitates the transport of acetyl CoA from the mitochondrial matrix to the cytoplasmic matrix. Within the matrix, acetyl CoA is initially condensed with oxaloacetic acid to form citric acid. Subsequently, citric acid is transported into the cytoplasmic matrix where it undergoes decomposition into acetyl CoA and oxaloacetic acid by citrate lyase, and the resulting acetyl CoA serves as a precursor for fatty acid synthesis (Inseok et al., 2021). Furthermore, a high concentration of CO_2_ leads to altered mRNA expression, improved synchronous expression patterns, and increased gene expression related to energy metabolism and light response, ultimately enhancing photosynthesis in algal cells (Figure 11).

The density of algal cells objectively reflects the growth of *E. gracilis*. UV mutagenesis combined with high carbon acclimation can change the adaptability of algal cells to the living environment to a certain extent, which is also verified in the physiological results of this study. It is found that the cell density, carbon sequestration rate, and photosynthetic pigment content of *E. gracilis* were increased after UV mutagenesis combined with high carbon acclimation. The results of physiological index data are mutually confirmed with the results obtained from the transcriptome. Thus, it can be inferred that certain algal cells possess robust repair capabilities enabling them not only to restore their normal state after UV mutagenesis and high carbon acclimation but also to undergo forward mutation during the repair process. This forward mutation confers resistance to a high carbon environment, resulting in enhanced photosynthetic activity as well as growth and reproductive capacity (Figure 11).

## 5 Conclusion

The objective of this study is to enhance the growth and carbon sequestration ability of *E. gracilis* under elevated CO_2_ concentration, as well as to reveal the physiological response mechanisms underlying carbon assimilation in a high-carbon environment, which may be of practical significance in the engineering application of reducing CO_2_ emissions. The findings and contributions of this study can be summarized as follows: (i) After UV mutagenesis and high carbon acclimation, the *E. gracilis* mutant, LE-ZW, exhibited enhanced cell growth and carbon sequestration capacity. Additionally, photosynthetic pigment content, photochemical efficiency, photosynthesis oxygen evolution and photosynthetic related enzyme activity increased, improvement in glycolysis and tricarboxylic acid cycling pathways, as well as enhanced ATP synthesis to support cell proliferation and carbon sequestration. (ii) The carbon assimilation capacity of the mutant was found to be higher at a 10% CO_2_ concentration compared to a 5% CO_2_ concentration, indicating its superior adaptability to high carbon environments. In response to elevated CO_2_ stress, multiple metabolic pathways were stimulated, leading to increased energy production and enhanced photosynthesis for efficient carbon fixation, thereby regulating cellular homeostasis in adaptation to environmental influences.

## Data Availability

The data presented in the study are deposited in the NCBI SRA repository, accession number PRJNA1150078.
