# Supplementary material for: Transcriptomic response analysis of ultraviolet mutagenesis combined with high carbon acclimation to promote photosynthetic carbon assimilation in Euglena gracilis
Source: Front Microbiol. 2024 Aug 29;15:1444420. doi: 10.3389/fmicb.2024.1444420 (PMC11390635; doi:10.3389/fmicb.2024.1444420)
Supplement: Supplementary file 1 [file Table_1.docx]

| Gene | Forward primer (F) | Reverse primer (R) |
| --- | --- | --- |
| *PsbQ* | GACACCGTGGCTTCCTTCA | GCGGCAACCTCGTCATAGA |
| *PsaD* | CAGCAAAACCTGCGTGGAC | GGCACCTCGGACAGTGCA |
| *PetD* | ATTTCTTCCCCACCTTCAACC | GCCAAGCCAGATAGCGACA |
| *PsbY* | AAGCATTCTGGCAGCATCTG | GGGCAGGCAACAAAATAAGTC |
