# Supplementary material for: Transcriptomic response analysis of ultraviolet mutagenesis combined with high carbon acclimation to promote photosynthetic carbon assimilation in Euglena gracilis
Source: Front Microbiol. 2024 Aug 29;15:1444420. doi: 10.3389/fmicb.2024.1444420 (PMC11390635; doi:10.3389/fmicb.2024.1444420)
Supplement: Supplementary file 2 [file Data_Sheet_1.docx]

>AF057161.1 *Euglena gracilis* actin mRNA, complete cds

CAAATGGCCGAAGAAATTGAACAACAAGCCTTAGTCTGTGACAATGGTTCAGGGATGGTTAAAGCTGGAT

TTGCAGGCGATGATGCTCCTCGCTGCGTCTTTCCCTCCATTGTGGGGCGCCGAAAAAACGACAGCGCAAT

GATGGGCACCGCAAAGAAAGATGCATACATTGGAGATGATGCACAGGCCAAGAGAGGAATTTTGTTCATC

AAGTATCCAATTGAGCACGGCATCGTGACCAACTGGGACGACATGGAGAAGATCTGGCATCACACTTTCT

TTAACGAACTGCGAGTTGCCCCGGAGGATCACCCCGTGCTTCTCACAGAGGCACCCATGAATCCCAAGAG

CAATCGTGAGAAGATGACCCAAATCATGTTTGAGACTTTCAATGTCCCAGCTCTTTATGTCAGCATTCAG

GCAGTTTTGTCCTTGTATTCCTCAGGACGAACAACTGGTATTGTTCTGGATTGTGGAGATGGTGTTTCTC

ACACAGTTCCCATCTACGAAGGTTATTCTCTCCCTCACGCTGTTCTGCGAATTGACATGGCTGGTCGGGA

TTTGACAGACTACATGATGAAATTGCTCACGGAACGTGGCCTCTCTTTCACAACAAGTGCTGAGAGAGAA

ATTGTGCGTGACGTCAAGGAGAAGCTGTGCTATGTGGCCTTGGATTTCGACGAGGAGATGTCCCTTGCAA

CTTCTTCTTCTTCTGTTGAAAAGGAGTACGAGTTGCCTGATGGAAACATCATTCAAGTGGGATCTGAGCG

TTTCCGTTGCCCGGAAGTGCTCATGAAGCCTTCCATGATTGGCCTTGAAGCCTGTGGTGTCCATGAAACC

ACCTTCAACTCCATCAACAAGTGCGACATTGATGTTCGCAAGGATTTGTACTCCAACATTGTGCTGTCTG

GTGGCACCACGATGTATGAGGGGCTTCCAGAGCGCATGTCCAAGGAGATCACGAACCTTGCGCCCAACAG

CATGAAGATCAAGGTGGTTGCACCCCCAGAAAGGAAGTACAGCGTGTGGATCGGCGGTTCCATCCTGGCC

TCCCTGTCCACATTCCAGAGCATGTGGATCAAGAAGGAGGAGTACGACGAGGCTGGCCCTGGCATTGTGC

ATCGCAAGTGCTTCTAGAGGCTGCCGCTGTCCTGCCCAGTCACGGGAGGTGCGAACCGGCCTGTGGGCCA

CTCAAATCCACCCCTCAGCGTGGGGCATCGGCAGTCGCTTTGCGCAAC

Forward primer (F): ATCTGAGCGTTTCCGTTGC

Reverse primer (R): GGAAGCCCCTCATACATCG

>CL792.Contig9_All CL792.Contig9_All

TATTTTTTTTCGACAGCAATGTCCATTGGTGATAAAAGCTTCGTTGTTTTGGCTGCCACCACTGCCTTAGCAGCTGGCGCGTTATTTGGCTTGGCTCTGAGCCCGTCGGTGGCGTCCAATTATGCCGCCCCCTCCGCCGTTCGTGCATCCACGACAATCTCTGCCGTCCGACCCGTGGTCTTCACAGGGTCGTACGTCCGTGGAGTCCAAACTGGACGCACCACGGTTGTGGAAGCCACTCCCAACCAGGAGGTGGAAACCCTGGCTTCTGCCAACAATCTTTGGGTGACAGCAGCAGCAGCCGGTCTGGCATTCAGCGCAGCCAGCTTGGTCCTGGCTTTGTTGCGGAAACCACGTTCAGAAGAGACCTATTCAATGCTTGCTGAAGCTGCTGAAGTGGGAAAAGCTCCTTGGACAGCACCAAAGCTGGAGGATGCCCTCAAAAATGAGAAGGCCCGGCAGAAGGCTGAAGCATTGAGCACCCAGCTGCCAGTTTTCTGGGACTTGCGGGACGCTTTGGGTGAGGTTCCCAAGTCGAAGTTCCCAGAAGTGTTTGCAAGGTACAAGGGGAGCACTGGTACTTTGCTGTCAGCAGCAAAAACCGAAGAATATTACGCCATCACTTGGACAAGTCCCAAGACCCAGATCTATGAGCTCCCAACAGGTGGCGCAGCAGAAATGGATGAGGGGTTGAACATCATGTATTTTGCCCGCAAGGAACAATGCCTTGCACTTGGAGCCCAGCTGCGCACAAAGTTCAAGCCCCGAATTGAGAGTTTCGCCATCTACCGTGTCTTCCCGAATGGGGAAACTCAGTACCTGCACCCGAAGGATGGCGTCTTCCCAGAGAAGGTCAACCAGGGCCGCACCAAGGCCAACCACAACCCCCGCAGCATTGGTGAGAACAAAGAGCCTGCTTCAGTGAAGTTCACTGGCACTACACCAAAGGATGTGCAGACTGAGGGCGCTGTGGCCATGTTTGCTGAAACTGGTGAGAAGGCAGCAAAACCTGCGTGGACCGTGCCCGATTTGGCTGATGCTTTGAAGAACACTTCAGCTGTTGCCAAGGCTAAGGGGATCGCCACCCAACTGCCCACTTTCTGGGAACTTCGGGATGCGCTGTCCGAGGTGCCCAAGTCCAAGTTCCCGGAGGTGTTCGCCAGTTACAAGGGGAGCACTGGCACCCTGCTTAGTGCGGCCGAGAAGGAAGAGAAGTACGTGATCACGTGGACCAGCCCCAAGAAGCAGATCTTCGAGCTGCCCACCGGTGGTGCAGCGGAGATGGAGGAGGGGGAGAACATCTTTTTCTTCGCCCGCAAGGAGCAGTGCCTGGCCCTGGGGGCCCAGCTGCGCAGCATTGGGGAGAATGCCGAGCCTGCCTCGGTGAAGTTCACAGGGACCACCCCCAAGGACGTCCAGACGGAGGGCGCTGTGGCCATGTTTGCCGAGACCGGCGAGAAGGCAGCAAAACCTGCGTGGACGGTGCCCAATCTGTCGGATGCTTTGAAGAACACAGCAGCTGTTGCCAAGGCGAAGGAGATCGCCACACAGTTGCCCACGTTCTGGGAACTTCGGGATGCACTGTCCGAGGTGCCCAAGTCCAAGAAGACGGCTTTCAAGCCCCGCATTGAGAACTTCCAGATCTTCCGGGTGTTCCCCAACGGGGAGGTGCAGTACCTGCACCCGAAGGACGGCGTCTTCCCTGAGAAGGTCAACCAAGGACGCACAAAGGCCAACCACAACCCCCGCAGCATCGGGGAGAATGCAGAGCCGGCCTCGGTGAAGTTCACCGGGACCACCCCCAAGGATGTGCAGACGGAGGGTGCTGTGGCTATGTTTTCTGAGACTGGTGAGAAGTTGGCAAAGTACAGCAAGTATGATGAAGAAATCCGGAAGGAGTTGGGACCCGTTGCCGAGAACAATTTTGTGATCTTCTGGGACAGCAACATTAACGGAACCCTCACGCTTCCAACAGGCGCAACCGCAGAAATGAAGGCAGGGCGGAATCGCTTCTGGTTTGCCACCGAAGAGCAGTGCTTGGCACTGAATGCGCAACTGCAGGACACCTTCAAGATCACTGATGCCTGGATCTGCGAAGTGACCCCCAATGGGGGCAAGCGATTGCACCCGAAGGAAGTTGCAGAGGGAGCAGTGGCGATGTTTGCTGAGACTGCCGTCAAGGACAGCAAGCCTGCTTGGCAAGTTCCGGATCTGGCGGATGCCCTGAAGAACGAGGCTGCAGTTCAGAAGGCCAAGGAGATCAGCACGCAGCTGCCGACTTTCTGGGAAGTGCGGGATGCTTTGTCCGAGGTGCCCAAGTCCCAGTTCCCGGAGGTGTTTGCCAATTTCAAGGGGAGCACTGGTACCCTGTTGGGAGCGGCTGAGAAGGAGGAGAAGTACGTGATTACCTGGACCAGCTCCAAGAAGCAGATCTTTGAGCTGCCCACAGGTGGTGCGGCGGAGATGGAGGAGGGGGAGAACATCTTTTATTTCTCCCGCAAGGAGCAGTGCCTGGCCCTGGGGGCCCAGCTGCGGACGGCTTTCAAGCCCCGCATTGAGAACTTCCAGATCTTCCGGGTGTTCCCCAACGGGGAGGTGCAGTACCTGCACCCAAAGGATGGCGTCTTCCCTGAGAAGGTCAACCAAGGACGCACAAAGGCCAACCACAACCCCCGCAGCATCGGGGAGAATGCAGAGCCGGCCTCGGTGAAGTTCACCGGGACCACCCCCAAGGATGTGCAGACGGAGGGTGCTGTGGCTATGTTTTCTGAGACTGGTGAGAAGTTGGCAAAGTACAGCAAGTATGATGAAGAAATCCGGAAGGAGTTGGGACCCGTTGCCGAGAACAATTTTGTGATCTTCTGGGACAGCAACATTAACGGAACCCTCACGCTTCCAACAGGCGCAACCGCAGAAATGAAGGCAGGGCGGAATCGCTTCTGGTTTGCCACCGAAGAGCAGTGCTTGGCACTGAATGCGCAACTGCAGGACACCTTCAAGATCACTGATGCCTGGATCTGCGAAGTGACCCCCAATGGGGGCAAGCGATTGCACCCGAAGGAAGTTGCAGAGGGAGCAGTGGCGATGTTTGCTGAGACTGCCGTCAAGGACAGCAAGCCTGCTTGGCAAGTTCCGGATCTGGCGGATGCCCTGAAGAACGAGGCTGCAGTTCAGAAGGCCAAGGAGATCAGCACGCAGCTGCCGACTTTCTGGGAAGTGCGGGATGCTTTGTCCGAGGTGCCCAAGTCCCAGTTCCCGGAGGTGTTTGCCAATTTCAAGGGGAGCACTGGTACCCTGTTGGGAGCGGCTGAGAAGGAGGAGAAGTAC

F:CAGCAAAACCTGCGTGGAC

R:GGCACCTCGGACAGTGCA

>Unigene1136_All Unigene1136_All

ATGTACGGTGAATCCCAGTCCTGGAGCGTTGGCATGCTGGCTGTTGCCGCTGTGTTGGGCTTCGTTGGTGGTGCCTCTCTGGGCCACCTGTACGCTGGTGCCAACACCAACCTGTACGTTGCAACCAACCAGCCAGTTGCAAATCGTGCGATGGCCGTCCGTGCCATGCCACGCAACGTGGAGGCTGTGATTCCTCAGACGGTCGCCGCTGCAAGCATTCTGGCAGCATCTGAGCCCGCCATGGCTGCTCAGCAGGTGGCCGCAGTTGCGGACAACCGCCTTGCTCTTGTTGGACTTATTTTGTTGCCTGCCCTCGGATGGGCTGCTTTCAACATCCTGAAGCCTGCTCTTCGCCAGTTGGAGAACATGCAAAAGAAGGCTGCTGGCCGGAGGTAA

F:AAGCATTCTGGCAGCATCTG

R:GGGCAGGCAACAAAATAAGTC

>CL5957.Contig1_All CL5957.Contig1_All

TCTATTTTTTTTCGCTTCAAAGGCACTTTCATAATGTCTGGAGACATGTCCACCCTGAAGCAGGTTGCAATCGTCGCCCCATGCTTTCTTCTGGGTCTTGTTGGAGCTTTGGTGATGACTGCACCCAACGCTGCTGTGCAGCACAACGTTCGGGCGCAACTTGTTGGTGCCAGTGTTGCTGCTGCTGCCATTGCCAACCCAGTATTTGCGGCACCCACCCCCATCGATGTCGCTGACTACCGCAACCAGCGTGAGGGATACGAGTACATTTACCAAGCTCGTGACCTGGACCTGGACGAGGATGTGCGCGCTGGATATGCCCAGGCACGTGATCCCTCCGTTGCCAAGGCTCGCATCAGCGAGTCCGTATCCCGTCTGATCAACCTGGGAAGTTTGGTGAAGAAGGCCTACTGGACTGCTGGTCGCGAGGAGCTGCGTGGCCAGGTTGGTACTCTGAGGTTCGACATCGACACCGTGGCTTCCTTCAAGTCCAGCAATGCTGAGAAGAAGGCACTCCTGGCAGCAAAGGCGGACTTGTTTACCGCTATTGACAAGCTTGACTTCGCCATGCGCAAGAAGGACCAGGCCAGCGCTTTGAAGTTCTATGACGAGGTTGCCGCTAAGGCAGCGGCCATCGCCAACGCTTGA

F:GACACCGTGGCTTCCTTCA

R:GCGGCAACCTCGTCATAGA

>CL3910.Contig1_All CL3910.Contig1_All

TCTATTTTTTTTCGCACGATGCAGAAAGCAGCTTTCGAGGATGTGGCGTTGACAACATACAACCCAGCCATGCTGCCACCAACTGGCACGCAACGCCTCCGTGGGGCGGCTGCTGTTGCGTGGGGATCCTTCCTATGGTTCGTTGCTGTGATGCTGGTGACCGCATGTGTCAGCCTGGGCTTCCAACGCTTCAGTGCTGATGCTTCTACCAATTTGATCAACACAATTGGGGCACGCTCTGTCTCGATGTCTTCCTCCCCCTCGAAGATGCCAAGCTTGGGCAATGTGGCACCACGAATTCAGACTGGTTACGTTGTTGCTGAGCCTGCTGAGAGTGCGTTGGAGCGGCGTTCAAGCTCTGTTGTGATGAACAATGTTATGAAGAAGCCTGATTTGTCTGACCCAAAGCTGCGGGCCAAGCTGGCCAAGGGAATGGGCCACAACTATTATGGTGAGCCAGCGTGGCCCAATGACCTCCTGTACATGTTCCCTGTTTGCATTCTTGGAACCTTTGCTGCAATTGTGGGTCTGGCTGTCATGCAGCCAACTCCAACTGGTGAGCCTGCAAACCCTTTCGCTACACCACTGGAGATCTTGCCTGAGTGGTATTTCTTCCCCACCTTCAACCTGCTGCGCGTGATCCCCAACAAGCTGCTGGGTGTACTGTCCATGGCAGCCGTCCCTGCTGGTCTGATCACAGTCCCTTTCATTGAGAACGTCAACAAATTCCAGAACCCTTTCCGTCGCCCTGTGGCCACAAGCGTGTTCCTTTTGGGCACTGTTGTCGCTATCTGGCTTGGCATTGGAGCAACCCTGCCAATTGACAAGGCTATCTCTCTGGGCTTCTGGTAA

F:ATTTCTTCCCCACCTTCAACC

R:GCCAAGCCAGATAGCGACA
